# 3-Chloro­anilinium 4-methyl­benzene­sulfonate

**DOI:** 10.1107/S1600536811041043

**Published:** 2011-10-12

**Authors:** Jerry P. Jasinski, James A. Golen, A. S. Praveen, H. S. Yathirajan, B. Narayana

**Affiliations:** a Department of Chemistry, Keene State College, 229 Main Street, Keene, NH 03435-2001, USA; b Department of Studies in Chemistry, University of Mysore, Manasagangotri, Mysore 570 006, India; c Department of Studies in Chemistry, Mangalore University, Mangalagangotri, 574 199, India

## Abstract

In the crystal structure of the title salt, C_6_H_7_ClN^+^·C_7_H_7_O_3_S^−^, the cations and anions are linked *via* N—H⋯O hydrogen bonds into doubled chains in [010]. Weak inter­molecular C—H⋯π inter­actions further link these chains into layers parallel to the *bc* plane.

## Related literature

For background to mol­ecular-ionic compounds, see: Czupinski *et al.* (2002 ▶); Katrusiak & Szafranski (2006 ▶). For related structures, see: Chanawanno *et al.* (2009 ▶); Chantrapromma *et al.* (2010 ▶); Collier *et al.* (2006 ▶); Fun *et al.* (2010 ▶); Li *et al.* (2005 ▶); Lin (2010 ▶); Tabatabaee & Noozari (2011 ▶); Wu *et al.* (2009 ▶). For normal bond lengths in organic compounds, see: Allen *et al.* (1987 ▶).
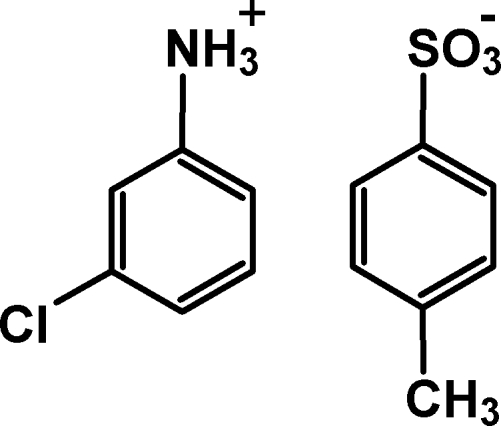


## Experimental

### 

#### Crystal data

C_6_H_7_ClN^+^·C_7_H_7_O_3_S^−^
*M* *_r_* = 299.76
Monoclinic, 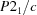

*a* = 12.7848 (3) Å
*b* = 6.7767 (2) Å
*c* = 16.1702 (4) Å
β = 105.081 (2)°
*V* = 1352.71 (6) Å^3^
*Z* = 4
Mo *K*α radiation
μ = 0.44 mm^−1^
*T* = 298 K
0.32 × 0.26 × 0.20 mm

#### Data collection

Oxford Diffraction Xcalibur Eos Gemini diffractometer
Absorption correction: multi-scan (*CrysAlis RED*; Oxford Diffraction, 2010 ▶) *T*_min_ = 0.872, *T*_max_ = 0.917
10965 measured reflections
3222 independent reflections
2723 reflections with *I* > 2σ(*I*)
*R*_int_ = 0.020

#### Refinement

*R*[*F* ^2^ > 2σ(*F* ^2^)] = 0.037
*wR*(*F* ^2^) = 0.109
*S* = 1.02
3222 reflections
182 parameters
6 restraints
H atoms treated by a mixture of independent and constrained refinement
Δρ_max_ = 0.38 e Å^−3^
Δρ_min_ = −0.47 e Å^−3^

### 

Data collection: *CrysAlis PRO* (Oxford Diffraction, 2010 ▶); cell refinement: *CrysAlis PRO*; data reduction: *CrysAlis RED* (Oxford Diffraction, 2010 ▶); program(s) used to solve structure: *SHELXS97* (Sheldrick, 2008 ▶); program(s) used to refine structure: *SHELXL97* (Sheldrick, 2008 ▶); molecular graphics: *SHELXTL* (Sheldrick, 2008 ▶); software used to prepare material for publication: *SHELXTL*.

## Supplementary Material

Crystal structure: contains datablock(s) global, I. DOI: 10.1107/S1600536811041043/cv5151sup1.cif

Structure factors: contains datablock(s) I. DOI: 10.1107/S1600536811041043/cv5151Isup2.hkl

Supplementary material file. DOI: 10.1107/S1600536811041043/cv5151Isup3.cml

Additional supplementary materials:  crystallographic information; 3D view; checkCIF report

## Figures and Tables

**Table 1:** Hydrogen-bond geometry (Å, °) *Cg*1 is the centroid of the C1–C6 ring.

*D*—H⋯*A*	*D*—H	H⋯*A*	*D*⋯*A*	*D*—H⋯*A*
N1—H1*NA*⋯O1^i^	0.92 (1)	1.86 (1)	2.7640 (19)	168 (2)
N1—H1*NC*⋯O3^ii^	0.93 (1)	1.87 (1)	2.7806 (18)	166 (2)
N1—H1*NB*⋯O2	0.93 (1)	1.92 (1)	2.8282 (19)	167 (2)
C12—H12*A*⋯*Cg*1^iii^	0.93	2.76	3.267 (2)	115

Symmetry codes: (i) 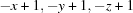; (ii) 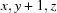; (iii) 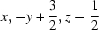.

**Fig. 1.**
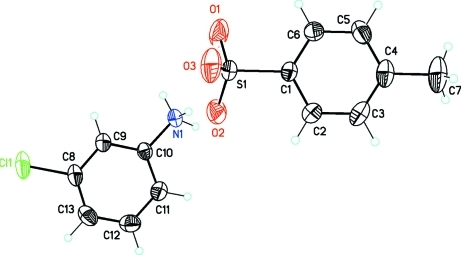
Molecular structure of the title compound showing the atom labeling scheme and 30% probability displacement ellipsoids.

## supplementary crystallographic information

### Comment

Recently much attention has been devoted to simple molecular-ionic crystals containing organic cations and anions due to the tunability of their special structural features and their interesting physical properties (Czupinski *et al.*, 2002; Katrusiak & Szafranski, 2006). A variety of pharmaceutical drugs are prepared as salts of benzenesulfonic acid and are known as besylates.  Crystal structures of some benzenesulfonate derivatives, viz., 2,4,6-triamino-1,3,5-triazin-1-ium 4-methylbenzenesulfonate monohydrate (Li *et al.*, 2005), ephedrine besylate (Collier *et al.*, 2006), 2-ethyl-6-methylanilinium 4-methylbenzenesulfonate (Wu *et al.*, 2009), 2-[(E)-2-(4-ethoxyphenyl)ethenyl]-1-methylpyridinium 4-methylbenzenesulfonate monohydrate (Chanawanno *et al.*, 2009), 2-aminopyrimidin-1-ium 4-methylbenzenesulfonate (Tabatabaee & Noozari, 2011), 4-(cyanomethyl)anilinium 4-methylbenzenesulfonate monohydrate (Lin, 2010), 1-methyl-2-[(E)-2-(2-thienyl)etheny] quinolinium 4-bromobenzenesulfonate (Fun *et al.*, 2010) and (E)-2-[4-(dimethylamino)styryl]-1-methylpyridinium 4-methylbenzenesulfonate monohydrate (Chantrapromma *et al.*, 2010) have been reported. In view of the importance of benzenesulphonic acid, we report herein the crystal structure of the title compound (I).

In the crystal structure of the title salt, C_6_H_7_ClN^+^. C_7_H_7_O_3_S^-^ (Fig. 1), the cations and anions are linked *via* N—H···O hydrogen bonds into doubled chains in [010].  Weak intermolecular C—H···π  interactions (Table 1) link further these chains into layers parallel to the *bc* plane.

### Experimental

To a stirred solution of 3-chloroaniline (0.67 g, 5.25 mmol) in methanol (15 ml), 4-methylbenzenesulfonic acid monohydrate (1g, 5.25 mmol ) was added, stirred at 323 K for 10 minutes and cooled to room temperature to afford the title compound (I). The single crystal was grown from methanol by slow evaporation method (M.P.: 538-540 K).

### Refinement

H1NA, H1NB and H1NC were located on a Fourier map and refined isotropically.  All of the remaining H atoms were placed in their calculated positions and then refined using the riding model with C—H bond lengths of 0.93Å (CH) or 0.96Å (CH_3_). Isotropic displacement parameters for these atoms were set to 1.18-1.20 (CH) or 1.49 (CH_3_) times *U*_eq_ of the parent atom.

### Crystal data

**Table:**

C_6_H_7_ClN^+^·C_7_H_7_O_3_S^−^	*F*(000) = 624
*M**_r_* = 299.76	*D*_x_ = 1.472 Mg m^−^^3^
Monoclinic, *P*2_1_/*c*	Mo *K*α radiation, λ = 0.71073 Å
Hall symbol: -P 2ybc	Cell parameters from 4281 reflections
*a* = 12.7848 (3) Å	θ = 3.2–30.0°
*b* = 6.7767 (2) Å	µ = 0.44 mm^−^^1^
*c* = 16.1702 (4) Å	*T* = 298 K
β = 105.081 (2)°	Block, colourless
*V* = 1352.71 (6) Å^3^	0.32 × 0.26 × 0.20 mm
*Z* = 4	

### Data collection

**Table:**

Oxford Diffraction Xcalibur Eos Gemini diffractometer	3222 independent reflections
Radiation source: Enhance (Mo) X-ray Source	2723 reflections with *I* > 2σ(*I*)
graphite	*R*_int_ = 0.020
Detector resolution: 16.1500 pixels mm^-1^	θ_max_ = 27.9°, θ_min_ = 3.3°
ω scans	*h* = −16→16
Absorption correction: multi-scan (*CrysAlis RED*; Oxford Diffraction, 2010)	*k* = −8→8
*T*_min_ = 0.872, *T*_max_ = 0.917	*l* = −21→18
10965 measured reflections	

### Refinement

**Table:**

Refinement on *F*^2^	Primary atom site location: structure-invariant direct methods
Least-squares matrix: full	Secondary atom site location: difference Fourier map
*R*[*F*^2^ > 2σ(*F*^2^)] = 0.037	Hydrogen site location: inferred from neighbouring sites
*wR*(*F*^2^) = 0.109	H atoms treated by a mixture of independent and constrained refinement
*S* = 1.02	*w* = 1/[σ^2^(*F*_o_^2^) + (0.0571*P*)^2^ + 0.4988*P*] where *P* = (*F*_o_^2^ + 2*F*_c_^2^)/3
3222 reflections	(Δ/σ)_max_ = 0.016
182 parameters	Δρ_max_ = 0.38 e Å^−^^3^
6 restraints	Δρ_min_ = −0.47 e Å^−^^3^

### Special details

**Table:**

Geometry. All esds (except the esd in the dihedral angle between two l.s. planes) are estimated using the full covariance matrix. The cell esds are taken into account individually in the estimation of esds in distances, angles and torsion angles; correlations between esds in cell parameters are only used when they are defined by crystal symmetry. An approximate (isotropic) treatment of cell esds is used for estimating esds involving l.s. planes.
Refinement. Refinement of *F*^2^ against ALL reflections. The weighted *R*-factor *wR* and goodness of fit *S* are based on *F*^2^, conventional *R*-factors *R* are based on *F*, with *F* set to zero for negative *F*^2^. The threshold expression of *F*^2^ > σ(*F*^2^) is used only for calculating *R*-factors(gt) *etc*. and is not relevant to the choice of reflections for refinement. *R*-factors based on *F*^2^ are statistically about twice as large as those based on *F*, and *R*- factors based on ALL data will be even larger.

### Fractional atomic coordinates and isotropic or equivalent isotropic displacement parameters (Å^2^)

**Table:**

	*x*	*y*	*z*	*U*_iso_*/*U*_eq_	
S1	0.71334 (3)	0.24308 (5)	0.57609 (2)	0.03033 (13)	
Cl1	0.48393 (5)	0.72176 (8)	0.12500 (3)	0.05637 (17)	
O1	0.60656 (10)	0.2751 (3)	0.58758 (10)	0.0679 (5)	
O2	0.74597 (11)	0.4022 (2)	0.52892 (8)	0.0533 (4)	
O3	0.72546 (12)	0.0520 (2)	0.54113 (9)	0.0589 (4)	
N1	0.61726 (11)	0.7273 (2)	0.45378 (9)	0.0314 (3)	
H1NC	0.6457 (12)	0.8338 (18)	0.4885 (10)	0.038*	
H1NB	0.6507 (12)	0.6166 (17)	0.4825 (10)	0.038*	
H1NA	0.5440 (7)	0.721 (2)	0.4477 (12)	0.038*	
C1	0.80291 (12)	0.2481 (2)	0.67983 (10)	0.0277 (3)	
C2	0.90883 (13)	0.3131 (2)	0.69108 (11)	0.0345 (3)	
H2A	0.9330	0.3532	0.6442	0.041*	
C3	0.97806 (13)	0.3175 (3)	0.77261 (12)	0.0403 (4)	
H3A	1.0489	0.3612	0.7800	0.048*	
C4	0.94414 (16)	0.2583 (2)	0.84357 (12)	0.0421 (4)	
C5	0.83782 (16)	0.1937 (3)	0.83099 (11)	0.0430 (4)	
H5A	0.8137	0.1532	0.8778	0.052*	
C6	0.76757 (14)	0.1887 (2)	0.74998 (10)	0.0356 (3)	
H6A	0.6967	0.1456	0.7426	0.043*	
C7	1.0204 (2)	0.2665 (3)	0.93256 (15)	0.0687 (7)	
H7A	1.0613	0.3869	0.9391	0.103*	
H7B	1.0690	0.1560	0.9406	0.103*	
H7C	0.9792	0.2618	0.9744	0.103*	
C8	0.58589 (15)	0.7473 (2)	0.21912 (10)	0.0347 (4)	
C9	0.56033 (13)	0.7206 (2)	0.29641 (10)	0.0312 (3)	
H9A	0.4907	0.6851	0.2983	0.037*	
C10	0.64148 (12)	0.7483 (2)	0.37058 (10)	0.0271 (3)	
C11	0.74566 (13)	0.7990 (2)	0.36897 (11)	0.0348 (3)	
H11A	0.7992	0.8178	0.4196	0.042*	
C12	0.76860 (15)	0.8212 (3)	0.29072 (12)	0.0412 (4)	
H12A	0.8387	0.8532	0.2888	0.049*	
C13	0.68918 (16)	0.7965 (2)	0.21532 (11)	0.0414 (4)	
H13A	0.7050	0.8128	0.1628	0.050*	

### Atomic displacement parameters (Å^2^)

**Table:**

	*U*^11^	*U*^22^	*U*^33^	*U*^12^	*U*^13^	*U*^23^
S1	0.0291 (2)	0.0332 (2)	0.0253 (2)	0.00048 (14)	0.00097 (14)	−0.00304 (13)
Cl1	0.0735 (4)	0.0580 (3)	0.0257 (2)	−0.0038 (2)	−0.0086 (2)	−0.00145 (18)
O1	0.0290 (7)	0.1261 (15)	0.0437 (8)	0.0078 (7)	0.0005 (6)	−0.0142 (8)
O2	0.0579 (8)	0.0538 (8)	0.0393 (7)	−0.0066 (6)	−0.0031 (6)	0.0160 (6)
O3	0.0771 (10)	0.0408 (7)	0.0450 (8)	0.0047 (7)	−0.0089 (7)	−0.0176 (6)
N1	0.0340 (7)	0.0355 (7)	0.0231 (6)	0.0001 (5)	0.0046 (5)	−0.0005 (5)
C1	0.0287 (7)	0.0251 (7)	0.0262 (7)	0.0009 (5)	0.0019 (6)	−0.0020 (5)
C2	0.0303 (8)	0.0347 (8)	0.0374 (8)	−0.0004 (6)	0.0068 (6)	−0.0022 (7)
C3	0.0297 (8)	0.0361 (8)	0.0480 (10)	0.0004 (7)	−0.0026 (7)	−0.0085 (7)
C4	0.0492 (10)	0.0313 (8)	0.0356 (9)	0.0063 (7)	−0.0073 (8)	−0.0051 (6)
C5	0.0594 (11)	0.0369 (9)	0.0302 (8)	−0.0026 (8)	0.0074 (8)	0.0037 (7)
C6	0.0378 (8)	0.0322 (8)	0.0353 (8)	−0.0061 (7)	0.0072 (7)	0.0015 (6)
C7	0.0792 (16)	0.0634 (14)	0.0436 (13)	0.0091 (11)	−0.0198 (11)	−0.0108 (9)
C8	0.0475 (9)	0.0283 (8)	0.0236 (7)	0.0016 (7)	0.0009 (6)	−0.0006 (5)
C9	0.0342 (8)	0.0300 (8)	0.0267 (7)	0.0002 (6)	0.0031 (6)	−0.0010 (6)
C10	0.0325 (7)	0.0239 (7)	0.0236 (7)	0.0025 (5)	0.0052 (6)	0.0008 (5)
C11	0.0345 (8)	0.0342 (8)	0.0329 (8)	−0.0021 (6)	0.0035 (6)	−0.0020 (6)
C12	0.0419 (9)	0.0392 (9)	0.0467 (10)	−0.0064 (7)	0.0189 (8)	−0.0009 (8)
C13	0.0625 (11)	0.0333 (8)	0.0320 (8)	−0.0050 (8)	0.0187 (8)	0.0015 (7)

### Geometric parameters (Å, °)

**Table:**

S1—O3	1.4374 (13)	C5—C6	1.382 (2)
S1—O2	1.4437 (13)	C5—H5A	0.9300
S1—O1	1.4413 (14)	C6—H6A	0.9300
S1—C1	1.7682 (15)	C7—H7A	0.9600
Cl1—C8	1.7359 (17)	C7—H7B	0.9600
N1—C10	1.464 (2)	C7—H7C	0.9600
N1—H1NC	0.928 (9)	C8—C9	1.383 (2)
N1—H1NB	0.927 (8)	C8—C13	1.379 (3)
N1—H1NA	0.916 (9)	C9—C10	1.379 (2)
C1—C6	1.386 (2)	C9—H9A	0.9300
C1—C2	1.390 (2)	C10—C11	1.382 (2)
C2—C3	1.384 (2)	C11—C12	1.379 (2)
C2—H2A	0.9300	C11—H11A	0.9300
C3—C4	1.387 (3)	C12—C13	1.379 (3)
C3—H3A	0.9300	C12—H12A	0.9300
C4—C5	1.392 (3)	C13—H13A	0.9300
C4—C7	1.515 (3)		
			
O3—S1—O2	112.92 (9)	C1—C6—C5	119.92 (16)
O3—S1—O1	112.90 (10)	C1—C6—H6A	120.0
O2—S1—O1	111.66 (10)	C5—C6—H6A	120.0
O3—S1—C1	106.32 (7)	C4—C7—H7A	109.5
O2—S1—C1	106.38 (7)	C4—C7—H7B	109.5
O1—S1—C1	106.03 (8)	H7A—C7—H7B	109.5
C10—N1—H1NC	109.7 (11)	C4—C7—H7C	109.5
C10—N1—H1NB	111.1 (11)	H7A—C7—H7C	109.5
H1NC—N1—H1NB	105.6 (14)	H7B—C7—H7C	109.5
C10—N1—H1NA	111.2 (12)	C9—C8—C13	121.74 (16)
H1NC—N1—H1NA	109.0 (13)	C9—C8—Cl1	118.62 (14)
H1NB—N1—H1NA	110.1 (13)	C13—C8—Cl1	119.64 (13)
C6—C1—C2	119.93 (14)	C10—C9—C8	117.81 (15)
C6—C1—S1	120.17 (12)	C10—C9—H9A	121.1
C2—C1—S1	119.91 (12)	C8—C9—H9A	121.1
C3—C2—C1	119.43 (16)	C9—C10—C11	121.86 (14)
C3—C2—H2A	120.3	C9—C10—N1	119.65 (14)
C1—C2—H2A	120.3	C11—C10—N1	118.47 (13)
C2—C3—C4	121.43 (16)	C12—C11—C10	118.70 (15)
C2—C3—H3A	119.3	C12—C11—H11A	120.7
C4—C3—H3A	119.3	C10—C11—H11A	120.7
C3—C4—C5	118.27 (16)	C13—C12—C11	120.97 (16)
C3—C4—C7	120.85 (19)	C13—C12—H12A	119.5
C5—C4—C7	120.9 (2)	C11—C12—H12A	119.5
C6—C5—C4	121.02 (17)	C12—C13—C8	118.91 (16)
C6—C5—H5A	119.5	C12—C13—H13A	120.5
C4—C5—H5A	119.5	C8—C13—H13A	120.5
			
O3—S1—C1—C6	−90.75 (15)	C2—C1—C6—C5	−0.2 (2)
O2—S1—C1—C6	148.65 (13)	S1—C1—C6—C5	−179.74 (13)
O1—S1—C1—C6	29.65 (16)	C4—C5—C6—C1	0.2 (3)
O3—S1—C1—C2	89.72 (14)	C13—C8—C9—C10	1.2 (2)
O2—S1—C1—C2	−30.89 (15)	Cl1—C8—C9—C10	−177.74 (11)
O1—S1—C1—C2	−149.88 (14)	C8—C9—C10—C11	−0.7 (2)
C6—C1—C2—C3	0.1 (2)	C8—C9—C10—N1	178.21 (13)
S1—C1—C2—C3	179.61 (12)	C9—C10—C11—C12	−0.4 (2)
C1—C2—C3—C4	0.1 (3)	N1—C10—C11—C12	−179.27 (15)
C2—C3—C4—C5	−0.1 (3)	C10—C11—C12—C13	1.0 (3)
C2—C3—C4—C7	−179.20 (16)	C11—C12—C13—C8	−0.6 (3)
C3—C4—C5—C6	−0.1 (3)	C9—C8—C13—C12	−0.5 (2)
C7—C4—C5—C6	179.06 (17)	Cl1—C8—C13—C12	178.34 (13)

### Hydrogen-bond geometry (Å, °)

**Table:**

Cg1 is the centroid of the C1–C6 ring.

**Table:**

*D*—H···*A*	*D*—H	H···*A*	*D*···*A*	*D*—H···*A*
N1—H1NA···O1^i^	0.92 (1)	1.86 (1)	2.7640 (19)	168.(2)
N1—H1NC···O3^ii^	0.93 (1)	1.87 (1)	2.7806 (18)	166.(2)
N1—H1NB···O2	0.93 (1)	1.92 (1)	2.8282 (19)	167.(2)
C12—H12A···Cg1^iii^	0.93	2.76	3.267 (2)	115

Symmetry codes: (i) −*x*+1, −*y*+1, −*z*+1; (ii) *x*, *y*+1, *z*; (iii) *x*, −*y*+3/2, *z*−1/2.
